# A taxonomic review of the *Neoserica* (sensu lato) *septemlamellata* group (Coleoptera, Scarabaeidae, Sericini)

**DOI:** 10.3897/zookeys.402.7360

**Published:** 2014-04-16

**Authors:** Dirk Ahrens, Wan-Gang Liu, Silvia Fabrizi, Ming Bai, Xing-Ke Yang

**Affiliations:** 1 Zoologisches Forschungsmuseum A. Koenig, Adenauerallee 160, 53113 Bonn, Germany; 2 Key Laboratory of Zoological Systematics and Evolution, Institute of Zoology, Chinese Academy of Sciences, Box 92, No. 1, Beichen West Road, Chaoyang District, Beijing, 100101, P.R. China; 3 University of Chinese Academy of Sciences, Yuquan Road, Shijingshan, Beijing, 100039, P.R. China

**Keywords:** Beetles, chafers, *Neoserica*, China, Laos, Vietnam, Thailand, new species

## Abstract

In the present paper the species belonging to the *Neoserica* (sensu lato) *septemlamellata* group, that included so far only four known species, are revised. Here we describe eleven new species originating mainly from Indochina and Southern China: *N. daweishanica* **sp. n.**, *N. gaoligongshanica* **sp. n.**, *N. guangpingensis* **sp. n.**, *N. igori* **sp. n.**, *N. jiulongensis* **sp. n.**, *N. plurilamellata* **sp. n.**, *N. weishanica* **sp. n.**, *N. yanzigouensis* **sp. n.** (China) *N. sapaensis* **sp. n.** (China, Vietnam), *N. bansongchana* **sp. n.**, *N. takakuwai* **sp. n.** (Laos). The lectotypes of *Neoserica septemlamellata* Brenske, 1898 and *N. septemfoliata* Moser, 1915 are designated. Keys to the species and species groups are given, the genitalia of all species and their habitus are illustrated and distribution maps are included.

## Introduction

The genus *Neoserica* Brenske, 1897 is with ca. 200 taxa one of the most species rich groups of Sericini. Since the redefinition of the genus (Pope 1960, Ahrens 2003) many other species so far grouped under *Neoserica* and being not directly related to the type species *Neoserica ursina* (Brenske, 1894) (i.e. *Neoserica* (sensu stricto) group; Ahrens 2003) are grouped preliminarily as *Neoserica* sensu lato (e.g. Ahrens 2004), a collective group that was identified to be neither related to *Neoserica* sensu stricto (Ahrens 2003) nor being monophyletic (Ahrens and Vogler 2008). They all await taxonomic revision based on which their relationships and classification can be subsequently established more rigorously.

In the current study we investigate the taxonomy of the representatives closely related to the species *Neoserica septemlamellata* Brenske, 1898, described originally from Myanmar. According to our present knowledge the species group is distributed from the eastern Himalaya to southern China and Indochina being mainly restricted to the higher elevated regions. The species of this group are characterised by a more or less distinctly tridentate protibia, by an antennal club composed of seven antennomeres in males and by a metafemur with a continuously serrated line adjacent to the anterior margin. However, all these features are likely to be homoplastic since they do occur also in other genera (e.g. in *Trioserica* Moser, 1922, *Nepaloserica* Frey, 1965, and *Lasioserica* Brenske, 1896, respectively). So far only four species were known to belong to this group, *Neoserica septemlamellata* Brenske, 1898, *Neoserica septemfoliata* Moser, 1915 as well as the recently described taxa *Neoserica changrae* Ahrens, 2004 and *Neoserica crenatolineata* Ahrens & Fabrizi, 2009. Here, eleven new species are described originating mainly from Indochina and Southern China.

## Material and methods

The terminology and methods used for measurements, specimen dissection and genital preparation follow Ahrens (2004). Data from specimens examined are cited in the text with original label contents given in quotation marks verbatim, multiple labels are separated by a “/”. Male genitalia were glued to a small pointed card and photographed in both lateral and dorsal view using a stereomicroscope Leica M125 with a Leica DC420C digital camera. In the automontage software as implemented in Leica Application Suite (V3.3.0) a number of single focussed images were combined in order to obtain an entirely focussed image. The resulting images were subsequently digitally edited.

Abbreviations used in the text for collection depositories are as follows:

CNAR Collection A. Naplov, Riga, Lettland;

CPPB Collection P. Pacholátko, Brno, Czech Republic;

HBUM Museum of Hebei University, Baoding, Hebei, China;

ISNB Institut Royal des Sciences naturelles de Belgique, Brüssel, Belgium;

IZAS Institute of Zoology, Chinese Academy of Sciences, Beijing, China;

MNHN Museum national d’Histoire naturelle, Paris, France;

NKU Nankai University, Tianjin, China;

NMPC National Museum (Natural History), Prague, Czech Republic;

ZFMK Zoologisches Forschungsinstitut und Museum A. Koenig, Bonn, Germany;

ZMHB Museum für Naturkunde Berlin, Germany;

ZSM Zoologische Staatssammlung, München, Germany.

### Key to species groups of *Neoserica* (sensu lato)

**Table:** 

1	Hypomeron not carinate	*Tetraserica* Ahrens, 2004
1’	Hypomeron carinate	2
2	Antennal club in female composed of 3 antennomeres	*Neoserica vulpes* group, *Neoserica lubrica* group, *Neoserica pilosula* group, *Neoserica calva* group, *Anomalophylla* Reitter, 1887, *Gynaecoserica* Brenske, 1896, *Leuroserica* Arrow, 1946, *Sericania* Motschulsky, 1860, *Calloserica* Brenske, 1894, *Lasioserica* Brenske, 1896, *Gastroserica* Brenske, 1897, *Neoserica* (s.str.) Brenske, 1894, *Trioserica* Moser, 1922, *Microserica* Brenske, 1894, *Oxyserica* Brenske, 1900, other *Neoserica* (s.l.)
2’	Antennal club in female composed of more than 3 antennomeres	3
3	Metatibia slender and long	4
3’	Metatibia short and wide	*Neoserica* (s. l.) *uniformis* group & *Neoserica multifoliata* group (from Indochina)
4	Antennal club of males with 7 antennomeres	5
4’	Antennal club of males with 7, 6 or less antennomeres	6
5	Metafemur with a continuously serrated line adjacent to the anterior margin of metafemur. Protibia more or less distinctly tridentate	*Neoserica septemlamellata* group
5’	Metafemur without a continuously serrated line adjacent to the anterior margin of metafemur. Protibia always distinctly bidentate	*Nepaloserica* Frey, 1965
6	Basis of labroclypeus dull. Antennal club of males with 6 or 7 antennomeres	7
6’	Antennal club of males with 5 or 4 antennomeres	8
7	Angle between basis of hypomeron and that of pronotum strongly rounded, angle of surfaces of hypomeron and pronotum basally blunt. Hypomeron basally strongly produced ventrally and transversely sulcate	*Lepidoserica* Nikolaev, 1979
7’	Angle between basis of hypomeron and that of pronotum sharp, angle of surfaces of hypomeron and pronotum sharp. Hypomeron basally not produced ventrally and not sulcate	*Neoserica abnormis* group
8	Body surface strongly shiny. Body small: 5,7–6,6 mm	*Neoserica speciosa* group
8’	Body surface dull. Body larger 8 mm	*Chrysoserica* Brenske, 1897

### *Neoserica septemlamellata* group - key to species (♂♂)

**Table:** 

1	Pronotum and elytra with dense, erect, long setae	2
1’	Pronotum and elytra nearly glabrous, without dense, erect, long setae	4
2	Left paramere basally extremely wide, without basal lobe	*Neoserica changrae* Ahrens
2’	Left paramere basally narrow, with a well developed basal lobe	3
3	Antennal club strongly reflexed and three times as long as the remaining antennomeres combined	*Neoserica weishanica* sp. n.
3’	Antennal club moderately reflexed and 2.5 times as long as the remaining antennomeres combined	*Neoserica septemlamellata* Brenske
4	Elytra shiny	5
4’	Elytra dull	9
5	Pronotum not or only weakly narrowed towards base	6
5’	Pronotum strongly narrowed towards base, and concavely sinuate before posterior angles. Phallobasis at apex ventrally produced	*Neoserica plurilamellata* sp. n.
6	Pronotum dull on disc	7
6’	Pronotum completely shiny	8
7	Phallobasis at apex ventrally not produced. Left paramere apically not widened. Basal tooth of protibia indistinct	*Neoserica gaoligongshanica* sp. n.
7’	Phallobasis at apex ventrally produced. Left paramere apically abruptly widened. Basal tooth of protibia distinct	*Neoserica jiulongensis* sp. n.
8	Left paramere (dorsal view) not widened basally (lateral view), at apex without ventral tooth	*Neoserica septemfoliata* Moser
8’	Left paramere (dorsal view) strongly widened basally (lateral view), at apex with a sharp ventral tooth	*Neoserica daweishanica* sp. n.
9	Antennal club shorter, 1.5 to 2 times as long as remaining antennomeres combined	10
9’	Antennal club longer, 2.5 to 3 times as long as remaining antennomeres combined	12
10	Basal lobe of left paramere directed medially. Basal tooth of protibia distinct	11
10’	Basal lobe of left paramere directed basally. Basal tooth of protibia indistinct	*Neoserica yanzigouensis* sp. n.
11	Right paramere narrow, nearly straight interiorly	*Neoserica bansongchana* sp. n.
11’	Right paramere wide, deeply sinuate interiorly at middle	*Neoserica guangpingensis* sp. n.
12	Parameres without any lobes, compact	13
12’	Parameres with lobes	14
13	Left paramere without triangular tooth laterally before apex	*Neoserica sapaensis* sp. n.
13’	Left paramere with a triangular tooth laterally before apex	*Neoserica igori* sp. n.
14	Basal lobe of left paramere directed distally, with a large convex distal lobe; right paramere with a small basal lobe. Protibia indistinctly tridentate, basal tooth small	*Neoserica crenatolineata* Ahrens & Fabrizi
14’	Basal lobe of left paramere directed medially, with a narrow and long distal lobe; right paramere without basal lobe. Protibia distinctly tridentate	*Neoserica takakuwai* sp. n.

## Systematics

### Neoserica (s.l.) septemlamellata

Brenske, 1898

http://species-id.net/wiki/Neoserica_septemlamellata

Figs 1A–D , 6

Neoserica septemlamellata Brenske, 1898: 349.
Aserica septemlamellata : Arrow 1946a: 14.

#### Type material examined.

Lectotype (here designated): 1 ♂ „Hte Birmanie Mines des Rubies 1200m 2300m Doherty 1890/ 7-lamellata type Brsk./ Museum Paris ex. Coll. R. Oberthür/ Type” (MNHN).

#### Additional material examined.

9 ex. „N-Thailand 20.–27.III.1990 Doi Ithang lg. Malicky” (ZSM), 7 ex. „N-Thailand 27.III.–3.IV.1990 Doi Ithanon lg. Malicky” (ZSM), 1 ex. „N-Thailand 14.–28.VIII.1990 Doi Ithanon lg. Malicky” (ZSM), 2 ex. „N-Thailand 10.–12.IV.1990 Doi Ithanon lg. Malicky” (ZSM), 1 ex. „N-Thailand 20.II.–6.III.1990 Doi Ithanon lg. Malicky” (ZSM), 9 ex. „N-Thailand 13.-20.III.1990 Doi Ithanon lg. Malicky” (ZSM), 3 ex. „NE-Laos: Hua Phan prov., Ban Saleui, Phou Pan (Mt.) - 20°12'N, 104°01'E; 14.iv.–15.v.2012; 1300–1900m; leg. C. Holzschuh Ankauf ZFMK Bonn 2012/13” (ZFMK), 16 ex. “Laos-NE Hua Phan prov., 20°12'N, 104°01'E, Phu Phan Mt., 1500–1900m, 17.5.–3.6.2007, leg. Vit Kuban” (ZFMK), 6 ex. “Laos-NE Hua Phan prov., 20°12'N, 104°01'E, Phu Phan Mt., 1500–1900m, 17.5.–3.6.2007, leg. C. Holzschuh” (ZFMK), 43 ex. “Laos-NE, Houa Phan prov., 20°13'09–19"N, 103°59'54"-104°00'03"E, 1480–1510m Phou Pane Mt., 22.IV.-14.V.2008 Vit Kuban leg. (NMPC), 1 ♂ “Mengzhe, Xishuangbanna, Yunnan, 23.VI.1958, 1700m, leg. Wang Shuyong” (IZAS).

#### Redescription.

Length: 8.3 mm, length of elytra: 6.2 mm, width: 5.2 mm. Body oblong, reddish brown, antennal club yellowish brown, labroclypeus shiny, dorsal surface dull, with dense and erect setae.

Labroclypeus trapezoidal, distinctly wider than long, widest at base, lateral margins weakly convex and strongly convergent anteriorly, anterior angles strongly rounded, anteriorly not sinuate medially, margins weakly reflexed; surface flat and shiny, basis without dull tomentum, punctation dense, small punctures mixed with coarse ones each bearing a long erect seta; frontoclypeal suture indistinctly incised, slightly elevated and distinctly angled medially; smooth area anterior to eye approximately 2.5 times as wide as long; ocular canthus moderately long (length = 1/3 of ocular diameter) and wide, with a few minute, superficial punctures and a few long setae. Frons dull, behind frontoclypeal suture shiny, with coarse and fine, dense punctures, densely covered with erect setae. Eyes moderately large, ratio diameter/interocular width: 0.64. Antenna with ten antennomeres, club with seven antennomeres, moderately reflexed, 2.5 times as long as remaining antennomeres combined; antennomere 4 subequal to half length of club, antennomere 3 half as long as pedicellus. Mentum elevated and slightly flattened anteriorly.

Pronotum subrectangular, widest shortly before base, lateral margins evenly convex and moderately convergent anteriorly, slightly narrowed basally, anterior angles sharp and distinctly produced, posterior angles blunt, slightly rounded at tip; anterior margin convexly produced medially, marginal line incomplete medially; surface densely and coarsely punctate, with long erect setae; setae of anterior and lateral border more robust and sparse; hypomeron distinctly carinate basally, carina not produced. Scutellum narrow and long, with coarse, dense punctures and a few short setae.

Elytra oblong, widest in posterior third, striae weakly impressed, finely and densely punctate, intervals weakly convex, finely and evenly densely punctate, third interval with punctures concentrated along striae, with dense, erect and long setae; epipleural edge wide, ending at widely rounded apical angle of elytra, epipleura densely setose, apical border narrowly membranous, with a fine fringe of microtrichomes (visible at 100×).

Ventral surface dull, coarsely and densely punctate, metasternum moderately setose; metacoxa glabrous, with a few long setae laterally, posterior margin straight; abdominal sternites finely and densely punctuate, minutely setose, with a transverse row of coarse punctures each bearing a robust, long seta. Mesosternum between mesocoxae half as wide as mesofemur. Ratio of length of metepisternum/metacoxa: 1/1.48. Pygidium strongly convex and dull, coarsely and densely punctate, with a narrow smooth midline, evenly covered with long erect setae.

Legs moderately slender and not very long; femora with two longitudinal rows of setae, finely and sparsely punctate, nearly glabrous; metafemur dull, anterior margin acute, immediately behind anterior edge with a continuously serrated line, punctures and setae of anterior longitudinal row complete, posterior margin in apical half ventrally smooth and not widened, posterior margin smooth dorsally, not serrated, with dense, short setae. Metatibia moderately slender and long, widest at apex, ratio of width/length: 1/2.9, dorsal margin sharply carinate, with three groups of spines, basal group shortly before middle, median one shortly behind middle, and apical group at 4/5 of metatibial length, basally with a few robust but single setae; beside dorsal margin with a continuously serrated line being subparallel with dorsal margin 4/5 of metatibial length; lateral face longitudinally convex, coarsely and densely punctate, in apical half punctures less dense, without convex subdorsal longitudinal carina on lateral face; ventral margin finely serrated, with four robust equidistant setae; medial face smooth, apex indistinctly sinuate interiorly near tarsal articulation. Tarsomeres with dense, short setae ventrally, not carinate laterally, smooth dorsally; metatarsomeres with a strongly serrated ventral ridge and a sharp subventral carina immediately beside it, first metatarsomere slightly shorter than following two tarsomeres combined and slightly longer than dorsal tibial spur. Protibia short, tridentate, basal tooth blunt; anterior claws symmetrical, basal tooth of inner claw sharply truncate at apex.

**Figure 1.**
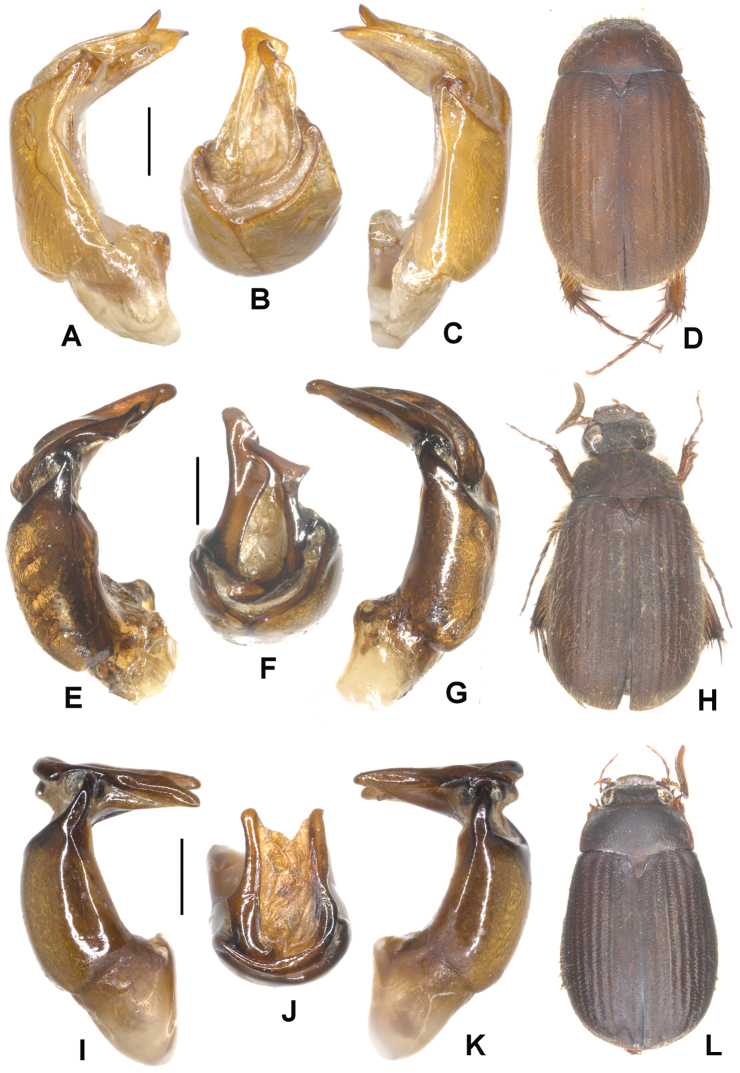
**A–D** *Neoserica septemlamellata* Brenske (Thailand: Doi Ithanon) **E–H** *Neoserica weishanica* Ahrens, Liu & Fabrizi sp. n. (holotype) **I–L** *Neoserica takakuwai* Ahrens, Liu & Fabrizi sp. n. (holotype) **A, E, I** Aedeagus, left side lateral view **C, G, K** Aedeagus, right side lateral view **B, F, J** parameres, dorsal view **D, H, L** Habitus. Scale: 0.5 mm.

Aedeagus: Fig. 1A–C.

#### Remarks.

The species was so far known only from Myanmar. Here it is first time recorded from Thailand and China. We designate here a lectotype for the species, since in the original description it is not mentioned on how many specimens it was based.

### Neoserica (s.l.) weishanica

Ahrens, Fabrizi & Liu sp. n.

http://zoobank.org/3336E93D-3093-4A31-A341-77C9B1A3C32B

http://species-id.net/wiki/Neoserica_weishanica

Figs 1E–H , 6

#### Type material examined.

Holotype ♂ “Yunnan 1800–500m 25.10N, 100.21E Weishan mts. 22.–25.6.92 David Kral lgt.” (ZFMK).

#### Description.

Length: 9.7 mm, length of elytra: 6.2 mm, width: 5.2 mm. Body oblong, reddish brown, antennal club yellowish brown, labroclypeus shiny, dorsal surface dull, with dense and erect setae.

Labroclypeus subtrapezoidal, slightly wider than long, widest at base, lateral margins weakly convex and strongly convergent anteriorly, anterior angles moderately rounded, anteriorly weakly sinuate medially, margins weakly reflexed; surface flat and shiny, basis without dull tomentum, punctation very dense, small punctures mixed with coarse ones each bearing a long erect seta; frontoclypeal suture distinctly incised, not elevated and distinctly angled medially; smooth area anterior to eye approximately 2.5 times as wide as long; ocular canthus moderately long (length = 1/3 of ocular diameter) and wide, with minute, dense punctures and a few long setae. Frons dull, behind frontoclypeal suture shiny, with coarse and fine, dense punctures, densely covered with long erect setae. Eyes large, ratio diameter/interocular width: 0.8. Antenna with ten antennomeres, club with seven antennomeres, strongly reflexed, three times as long as remaining antennomeres combined; antennomere 4 subequal to half length of club, antennomere 3 half as long as pedicellus. Mentum elevated and slightly flattened anteriorly.

Pronotum subrectangular, widest at base, lateral margins in basal half straight and subparallel, convex and moderately convergent in anterior half, anterior angles moderately sharp and distinctly produced, posterior angles blunt, slightly rounded at tip; anterior margin convexly produced medially, marginal line incomplete medially; surface densely and coarsely punctate, with long erect setae; setae of anterior and lateral border more robust and sparse; hypomeron distinctly carinate basally, carina not produced. Scutellum narrow and long, with coarse, dense punctures and a few short setae.

Elytra oblong, widest in posterior third, striae weakly impressed, finely and densely punctate, intervals weakly convex, finely and evenly densely punctate, third interval with punctures concentrated along striae, with dense, erect and long setae; epipleural edge wide, ending at widely rounded apical angle of elytra, epipleura densely setose, apical border narrowly membranous, with a fine fringe of microtrichomes (visible at 100×).

Ventral surface dull, coarsely and densely punctate, metasternum densely setose; metacoxa glabrous, with numerous long setae laterally, posterior margin straight; abdominal sternites finely and densely punctuate, minutely setose, with a transverse row of coarse punctures each bearing a robust, long seta. Mesosternum between mesocoxae half as wide as mesofemur. Ratio of length of metepisternum/metacoxa: 1/1.41. Pygidium weakly convex and dull, coarsely and densely punctate, with a narrow smooth midline, evenly covered with long erect setae.

Legs moderately slender and not very long; femora with two longitudinal rows of setae, finely and moderately densely punctate, nearly glabrous; metafemur dull, anterior margin acute, immediately behind anterior edge with a continuously serrated line, punctures and setae of anterior longitudinal row complete, posterior margin in apical half ventrally smooth and not widened, posterior margin smooth dorsally, not serrated, with dense, short setae. Metatibia moderately slender and long, widest at apex, ratio of width/ length: 1/2.8, dorsal margin sharply carinate, with three groups of spines, basal group shortly before middle, median one at two thirds, and apical group at 4/5 of metatibial length, basally with a few robust but single setae; beside dorsal margin with a continuously serrated line being subparallel with dorsal margin 4/5 of metatibial length and ending at apex of metatibia; lateral face longitudinally convex, coarsely and densely punctate, in apical half punctures less dense, without convex subdorsal longitudinal carina on lateral face; ventral margin finely serrated, with four robust equidistant setae; medial face smooth, apex indistinctly sinuate interiorly near tarsal articulation. Tarsomeres with dense, short setae ventrally, laterally not carinate, dorsally with a few coarse punctures each bearing a robust short seta at posterior part of basal two tarsomeres; metatarsomeres with a strongly serrated ventral ridge and a sharp subventral carina immediately beside it, first metatarsomere slightly shorter than following two tarsomeres combined and slightly longer than dorsal tibial spur. Protibia short, tridentate, basal tooth blunt; anterior claws symmetrical, basal tooth of inner claw sharply truncate at apex.

Aedeagus: Fig. 1E–G. Female unknown.

#### Diagnosis.

The new species is in genital and external morphology very similar to *Neoserica septemlamellata*. It differs by the significantly longer and strongly reflexed club (male) and the dorsal punctures on meso- and metatarsomeres as well as by shape of parameres: the left paramere (both species share the long basal lobe directed mesally) has at middle a sharp triangular tooth laterally, the basal lobe is flat in cross section, the right paramere is not widened at apex showing, however, a blunt median extension a third before the apex.

#### Etymology.

The new species is named according to its type locality, Weishan mountains.

### Neoserica (s.l.) takakuwai

Ahrens, Fabrizi & Liu sp. n.

http://zoobank.org/44F563D7-9321-4EF0-970C-B8C3954DA463

http://species-id.net/wiki/Neoserica_takakuwai

Figs 1I–L , 7

#### Type material examined.

Holotype ♂ “Mt. Phu Pan, Xam Neua, Houapan, N. Laos, 19–21.V.2005 Takakuwa, M. leg.” (ZFMK). Paratypes: 1 ♂ “Laos-NE Hua Phan prov., 20°12'N, 104°01'E, Phu Phan Mt., 1500–1900m, 17.5.–3.6.2007, leg. Vit Kuban” (NMPC), 2 ♂♂ “Laos-NE Hua Phan prov., 20°12'N, 104°01'E, Phu Phan Mt., 1500-1900m, 17.5.-3.6.2007, leg. C. Holzschuh” (ZFMK), 3 ♂♂ “Laos-NE, Houa Phan prov., 20°13'09-19"N, 103°59'54"–104°00'03"E, 1480–1510m Phou Pane Mt., 22.IV.–14.V.2008 Vit Kuban leg.” (ZFMK), 1 ♂, 1 ♀ “Laos-NE, Houa Phan prov., 20°13'09–19"N, 103°59'54"–104°00'03"E, 1480–1510m Phou Pane Mt., 22.4.–14.5.2008 Vit Kuban leg.” (ZFMK).

#### Description.

Length: 8.5 mm, length of elytra: 5.8 mm, width: 4.9 mm. Body oblong, dark brown, ventral surface and legs reddish brown, antennal club yellowish brown, dorsal surface dull, nearly glabrous except a few long setae on head.

Labroclypeus short and trapezoidal, distinctly wider than long, widest at base, lateral margins nearly straight and strongly convergent anteriorly, anterior angles strongly rounded, anterior margin very weakly sinuate medially, margins moderately reflexed; surface flat and shiny, basis without dull tomentum, punctation dense, small punctures mixed with coarse ones each bearing a long erect seta; frontoclypeal suture distinctly incised, not elevated and distinctly angled medially; smooth area anterior to eye approximately 2.5 times as wide as long; ocular canthus moderately long (length = 1/3 of ocular diameter) and wide, with minute punctures and a few long setae. Frons dull, anterior quarter shiny, with coarse and fine, dense punctures, densely covered with long erect setae. Eyes large, ratio diameter/interocular width: 0.74. Antenna with ten antennomeres, club with seven antennomeres, moderately reflexed, 2.5 times as long as remaining antennomeres combined; antennomere 4 slightly longer than half of club length, antennomere 3 half as long as pedicellus. Mentum elevated and slightly flattened anteriorly.

Pronotum moderately transverse, subrectangular, widest at base, lateral margins evenly weakly convex and moderately convergent towards moderately sharp and distinctly produced anterior angles, posterior angles blunt, slightly rounded at tip; anterior margin moderately convexly produced medially, with a broadly incomplete marginal line medially; surface densely and moderately coarsely punctate, only with minute setae; setae of anterior and lateral border fine and dense; hypomeron distinctly carinate basally, carina not produced. Scutellum narrow and long, dull, with fine, dense punctures also on midline, with minute setae.

Elytra oblong, widest at posterior third, striae weakly impressed, finely and densely punctate, intervals weakly convex, finely and densely punctate, punctures concentrated along striae, odd intervals with single long erect setae, otherwise punctures only with very minute setae; epipleural edge wide, ending at widely rounded apical angle of elytra, epipleura densely setose, apical border narrowly membranous, with a fine fringe of microtrichomes (visible at 100×).

Ventral surface dull, coarsely and densely punctate, metasternum moderately setose; metacoxa glabrous, with a few long setae laterally, posterior margin straight; abdominal sternites finely and densely punctuate, minutely setose, with a transverse row of coarse punctures each bearing a robust, long seta. Mesosternum between mesocoxae half as wide as mesofemur. Ratio of length of metepisternum/metacoxa: 1/1.56. Pygidium moderately convex and dull, coarsely and densely punctate, without impunctate midline, with dense, long, erect and minute setae on apical half.

Legs moderately slender and not very long; femora with two longitudinal rows of setae, finely and moderately densely punctate, nearly glabrous; metafemur dull, anterior margin acute, immediately behind anterior edge with a continuously serrated line, punctures and setae of anterior longitudinal row complete, posterior margin in apical half ventrally smooth and not widened, posterior margin smooth dorsally, not serrated, with dense, short setae. Metatibia moderately slender and long, widest at apex, ratio of width/length: 1/2.9; dorsal margin sharply carinate, with three groups of robust spines, basal group shortly before middle, median one at two thirds, and apical group at 4/5 of metatibial length, basally with a few robust but single setae; beside dorsal margin with a continuously serrated line being convergent with dorsal margin 4/5 of metatibial length; lateral face longitudinally convex, finely and moderately densely punctate, without convex subdorsal longitudinal carina on lateral face; ventral margin finely serrated, with four robust, nearly equidistant setae; medial face smooth, apex indistinctly sinuate interiorly near tarsal articulation. Tarsomeres with dense, short setae ventrally, not carinate laterally, smooth dorsally; metatarsomeres with a strongly serrated ventral ridge and a sharp subventral carina immediately beside it, first metatarsomere slightly shorter than following two tarsomeres combined and slightly longer than dorsal tibial spur. Protibia short, tridentate, basal tooth distinct; anterior claws symmetrical, basal tooth of inner claw sharply truncate at apex.

Aedeagus: Fig. 1I–K.

#### Diagnosis.

The new species is in shape of parameres somewhat similar to *Neoserica weishanica* sp. n., but it differs by having the parameres straight, lacking a external blunt tooth to the left paramere, and a median internal extension to the right one.

#### Etymology.

The new species is named after the first collector of this species, M. Takakuwa.

#### Variation.

Length: 8.5–9.3 mm, length of elytra: 5.8–7.5 mm, width: 4.9–5.8 mm. Female: Antennal club composed of five antennomeres, slightly longer than remaining antennomeres combined, first joint of club a quarter of length of club, 5th antennomere slightly transversely produced; eyes slightly smaller than in male (ratio diameter/interocular width: 0.56).

### Neoserica (s.l.) yanzigouensis

Ahrens, Fabrizi & Liu sp. n.

http://zoobank.org/8271DC13-1B45-4F80-ADDF-10CE14EA67DE

http://species-id.net/wiki/Neoserica_yanzigouensis

Figs 2A–D , 7

#### Type material examined.

Holotype ♂ “Yanzigou, Xinxing, Luding, Sichuan, 7.VIII.2004, 1560m, leg. Bai Ming, Wan Xia” (IZAS). Paratypes. 1 ♂ “Yanzigou, Xinxing, Luding, Sichuan, 7.VIII.2004, 1560m, leg. Bai Ming, Wan Xia” (IZAS), 6 ♂♂ “Yanzigou, Xinxing, Luding, Sichuan, 7.VIII.2004, 1500m, leg. Zhang Yong” (IZAS, ZFMK), 2 ♂♂ “Yanzigou, Xinxing, Luding, Sichuan, 7.VIII.2004, 1560m, leg. Bai Ming “ (IZAS).

#### Description.

Length: 8.2 mm, length of elytra: 6.4 mm, width: 4.6 mm. Body oblong, dark reddish brown, antennal club yellowish brown, dorsal surface dull, nearly glabrous except a few long setae on head.

Labroclypeus trapezoidal, distinctly wider than long, widest at base, lateral margins nearly straight and strongly convergent anteriorly, anterior angles strongly rounded, anterior margin weakly sinuate medially, margins moderately reflexed; surface flat and shiny, basis without dull tomentum, punctation dense, small punctures mixed with coarse ones each bearing a long erect seta; frontoclypeal suture distinctly incised, not elevated and distinctly angled medially; smooth area anterior to eye approximately 2.5 times as wide as long; ocular canthus moderately long (length = 1/3 of ocular diameter) and wide, with minute punctures and a few long setae. Frons dull, anterior quarter shiny, with coarse and fine, dense punctures, densely covered with long erect setae. Eyes large, ratio diameter/interocular width: 0.74. Antenna with ten antennomeres, club with seven antennomeres, moderately reflexed, twice as long as remaining antennomeres combined; antennomere 4 subequal to half length of club, antennomere 3 half as long as pedicellus. Mentum elevated and slightly flattened anteriorly.

Pronotum moderately transverse, subrectangular, widest at base, lateral margins weakly convex and moderately convergent towards moderately sharp and distinctly produced anterior angles, posterior angles blunt, slightly rounded at tip; anterior margin convexly produced medially, marginal line incomplete medially; surface densely and coarsely punctate, only with minute setae; setae of anterior and lateral border fine and sparse; hypomeron distinctly carinate basally, carina not produced. Scutellum narrow and long, dull, with fine, dense punctures and minute setae.

Elytra oblong, widest in posterior third, striae weakly impressed, finely and densely punctate, intervals weakly convex, finely and densely punctate, punctures concentrated along striae, with minute setae, odd intervals with a few erect setae (partially lacking on disc); epipleural edge wide, ending at widely rounded apical angle of elytra, epipleura densely setose, apical border narrowly membranous, with a fine fringe of microtrichomes (visible at 100×).

Ventral surface dull, coarsely and densely punctate, metasternum moderately setose; metacoxa glabrous, with a few long setae laterally, posterior margin straight; abdominal sternites finely and densely punctuate, minutely setose, with a transverse row of coarse punctures each bearing a robust, long seta. Mesosternum between mesocoxae half as wide as mesofemur. Ratio of length of metepisternum/metacoxa: 1/1.41. Pygidium strongly convex and dull, coarsely and densely punctate, with a narrow smooth midline, with dense, long erect setae and minute setae on apical half.

Legs moderately slender and not very long; femora with two longitudinal rows of setae, finely and moderately densely punctate, nearly glabrous; metafemur dull, anterior margin acute, immediately behind anterior edge with a continuously serrated line, punctures and setae of anterior longitudinal row complete, posterior margin in apical half ventrally smooth and not widened, posterior margin smooth dorsally, not serrated, with dense, short setae. Metatibia moderately slender and long, widest at apex, ratio of width/length: 1/3.2; dorsal margin sharply carinate, with three groups of spines, basal group shortly before middle, median one shortly behind middle, and apical group at 4/5 of metatibial length, basally with a few robust but single setae; beside dorsal margin with a continuously serrated line being subparallel with dorsal margin 4/5 of metatibial length; lateral face longitudinally convex, finely and moderately densely punctate, without convex subdorsal longitudinal carina on lateral face; ventral margin finely serrated, with four robust equidistant setae; medial face smooth, apex indistinctly sinuate interiorly near tarsal articulation. Tarsomeres with dense, short setae ventrally, not carinate laterally, smooth dorsally; metatarsomeres with a strongly serrated ventral ridge and a sharp subventral carina immediately beside it, first metatarsomere slightly shorter than following two tarsomeres combined and slightly longer than dorsal tibial spur. Protibia short, tridentate, but basal tooth rather indistinct; anterior claws symmetrical, basal tooth of inner claw sharply truncate at apex.

**Figure 2.**
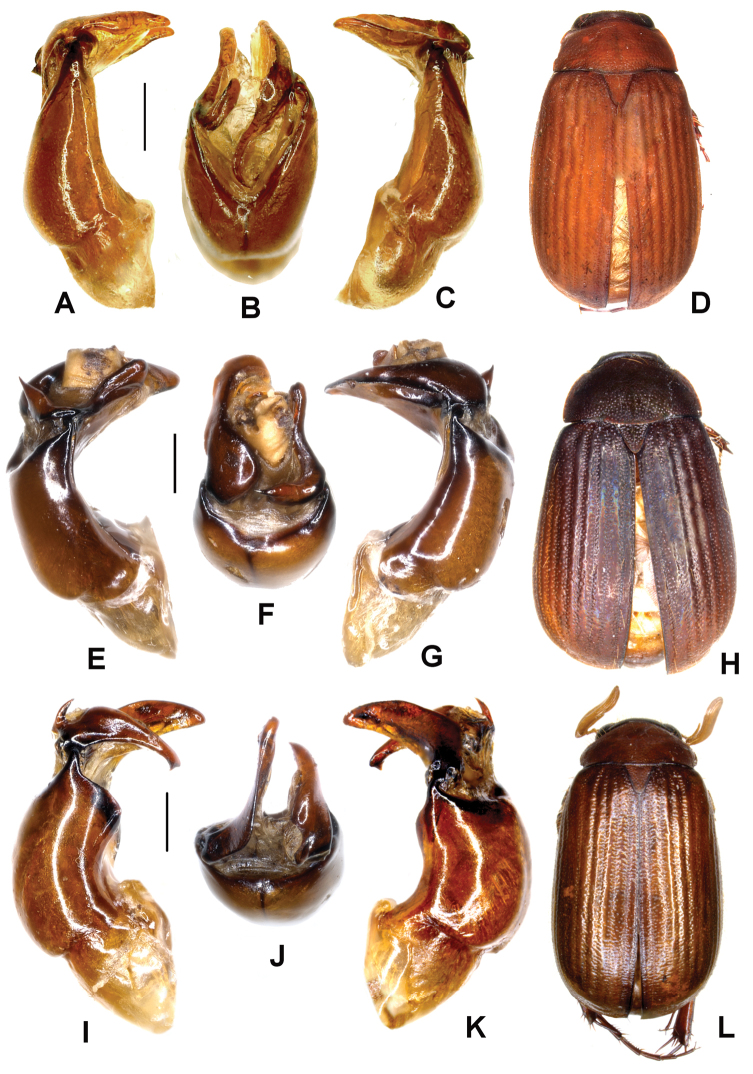
**A–D** *Neoserica yanzigouensis* Ahrens, Liu & Fabrizi sp. n. (holotype) **E–H** *Neoserica guangpingensis* Ahrens, Liu & Fabrizi sp. n. (holotype) **I–L** *Neoserica plurilamellata* Ahrens, Liu & Fabrizi sp. n. (holotype) **A, E, I** Aedeagus, left side lateral view **C, G, K** Aedeagus, right side lateral view **B, F, J** parameres, dorsal view **D, H, L** Habitus. Scale: 0.5 mm.

Aedeagus: Fig. 2A–C. Female unknown.

#### Diagnosis.

The new species is similar to *Neoserica takakuwai* sp. n. in external morphology. It may be distinguished by its more reddish brown colour, by the slightly shorter antennal club being twice as long as remaining antennomeres combined, as well as by the basal lobe of the left paramere directed basally (and not medially as in *Neoserica takakuwai* sp. n.).

#### Etymology.

The new species is named after its type locality, Yanzigou.

#### Variation.

Length: 8.2–9.6 mm, length of elytra: 6.4–7.1 mm, width: 4.6–5.2 mm.

### Neoserica (s.l.) guangpingensis

Ahrens, Fabrizi & Liu sp. n.

http://zoobank.org/17E90087-D9C9-41EC-8EF7-593A14006B5A

http://species-id.net/wiki/Neoserica_guangpingensis

Figs 2E–H , 7

#### Type material examined.

Holotype: ♂ “839462 Neoserica spYU09_2 China S. Murzin 29.07.2009 Guang Ping, 34km N Jihong Yunnan Pr. Xichuanbanna 1200m” (ZFMK).

#### Description.

Length: 8.8 mm, length of elytra: 6.5 mm, width: 5.2 mm. Body oblong, dark brown, antennal club yellowish brown, dorsal surface dull, except a few long setae on head and sides of elytra nearly glabrous.

Labroclypeus subtrapezoidal, considerably wider than long, widest at base, lateral margins moderately convex and strongly convergent anteriorly, anterior angles strongly rounded, anterior margin weakly sinuate medially, margins moderately reflexed; surface convexly elevated medially and shiny, basis without dull tomentum, punctation dense, small punctures mixed with coarse ones each bearing a long erect seta; frontoclypeal suture distinctly incised, not elevated and distinctly angled medially; smooth area anterior to eye approximately 2.5 times as wide as long; ocular canthus short (length = 1/4 of ocular diameter) and wide, with fine, moderately dense punctures and a few long setae. Frons in posterior quarter dull, otherwise moderately shiny, with coarse and fine, dense punctures, densely covered with long erect setae being partly bent posteriorly. Eyes moderately large, ratio diameter/interocular width: 0.61. Antenna with ten antennomeres, club with seven antennomeres, moderately reflexed, twice as long as remaining antennomeres combined; antennomere 4 subequal to half of length of club, antennomere 3 half as long as pedicellus. Mentum elevated and slightly flattened anteriorly.

Pronotum transverse, widest shortly before base, lateral margins evenly convex and moderately convergent anteriorly, anterior angles moderately sharp and distinctly produced, posterior angles strongly rounded; anterior margin moderately convexly produced medially, with a medially widely incomplete marginal line; surface densely and coarsely punctate, only with minute setae, and a few long erect setae on anterior sides; setae of lateral border fine and moderately dense, those of anterior margin numerous and in part widely displaced from margin; hypomeron distinctly carinate basally, carina not produced. Scutellum narrow and long, dull, with fine, dense punctures, on midline impunctate, with minute setae.

Elytra oblong, widest in posterior third, striae weakly impressed, finely and densely punctate, intervals weakly convex, finely and densely punctate, punctures mainly concentrated along striae, with minute setae, odd intervals on sides and apical declivity with a few erect setae (lacking on disc); epipleural edge wide, ending at widely rounded apical angle of elytra, epipleura densely setose, apical border broadly membranous, with a fine fringe of microtrichomes (visible at 100×).

Ventral surface dull, coarsely and densely punctate, metasternum moderately setose; metacoxa glabrous, with a few long setae laterally, posterior margin straight; abdominal sternites finely and densely punctuate, minutely setose, with a transverse row of coarse punctures each bearing a robust, long seta. Mesosternum between mesocoxae half as wide as mesofemur. Ratio of length of metepisternum/metacoxa: 1/1.62. Pygidium strongly convex and dull, coarsely and densely punctate, with a narrow smooth midline, with dense and long erect setae and minute setae.

Legs moderately slender and not very long; femora with two longitudinal rows of setae, finely and moderately densely punctate between rows, nearly glabrous; metafemur dull, anterior margin acute, immediately behind anterior edge with a continuously serrated line, punctures and setae of anterior longitudinal row complete, posterior margin in apical half ventrally smooth and little widened and smooth, posterior margin smooth dorsally, not serrated, with dense, short setae. Metatibia moderately slender and short, widest at apex, ratio of width/length: 1/2.6; dorsal margin sharply carinate, with three groups of spines, basal group at the middle, median one at two thirds, and apical group at 4/5 of metatibial length, basally with a few robust but single setae; beside dorsal margin with a continuously serrated line being subparallel with dorsal margin 4/5 of metatibial length and ending shortly before metatibial apex; lateral face longitudinally convex, coarsely and moderately densely punctate, with a few robust setae, in apical half punctures less dense, without convex subdorsal longitudinal carina on lateral face; ventral margin finely serrated, with four robust, nearly equidistant setae; medial face smooth, apex indistinctly sinuate interiorly near tarsal articulation. Tarsomeres with dense, short setae ventrally, not carinate laterally, smooth dorsally; metatarsomeres with a strongly serrated ventral ridge and a sharp subventral carina immediately beside it, first metatarsomere as long as following two tarsomeres combined and slightly longer than dorsal tibial spur. Protibia short, distinctly tridentate; anterior claws symmetrical, basal tooth of inner claw sharply truncate at apex.

Aedeagus: Fig. 2E–G.

#### Diagnosis.

*Neoserica guangpingensis* sp. n. differs from very similar *Neoserica takakuwai* sp. n. by the wide right paramere being deeply and concavely sinuate internally.

#### Etymology.

The new species is named according to its type locality Guang Ping (China).

### Neoserica (s.l.) plurilamellata

Ahrens, Fabrizi & Liu sp. n.

http://zoobank.org/DA6AA1B1-AFE0-4BAB-8104-2FC24EBB4EFF

http://species-id.net/wiki/Neoserica_plurilamellata

Figs 2I–L , 7

#### Type material examined.

Holotype: ♂ “China, N.Yunnan, env. Xiaguan, 2400m, 29.vii.2002, leg. S. Murzin, I. Shokhin/ Coll. P. Pacholátko Brno Merhautova 68 Czech Republic/ 858 Sericini: Asia spec.” (CPPB). Paratype: 1 ♂ “Pantian’ge, Weixi, Yunnan, 23.VII.1981, 2500m, light trap, leg. Liao Subai” (IZAS).

#### Description.

Length: 8.3 mm, length of elytra: 7.0 mm, width: 5.2 mm. Body oblong, reddish brown, antennal club yellowish brown, dorsal surface moderately shiny, nearly glabrous except a few long setae on head.

Labroclypeus trapezoidal, distinctly wider than long, widest at base, lateral margins straight and strongly convergent anteriorly, anterior angles moderately rounded, anteriorly weakly sinuate medially, margins moderately reflexed; surface slightly convex and shiny, basis without dull tomentum, punctation dense, small punctures mixed with coarse ones each bearing a long erect seta; frontoclypeal suture indistinctly incised, not elevated and distinctly angled medially; smooth area anterior to eye approximately 2.5 times as wide as long; ocular canthus moderately long (length = 1/3 of ocular diameter) and wide, with a few minute punctures and a few long setae. Frons shiny, with coarse and fine, dense punctures, densely covered with long erect setae. Eyes large, ratio diameter/interocular width: 0.82. Antenna with ten antennomeres, club with seven antennomeres, moderately reflexed, 2.5 times as long as remaining antennomeres combined; antennomere 4 subequal to two thirds of length of club, antennomere 3 half as long as pedicellus. Mentum elevated and slightly flattened anteriorly.

Pronotum subrectangular, widest at middle, lateral margins strongly and evenly convex, distinctly convergent anteriorly and posteriorly, before posterior angles deeply concavely sinuate, anterior angles sharp and moderately produced, posterior angles right-angled, very weakly rounded at tip; anterior margin convexly produced medially, marginal line incomplete medially; surface densely and coarsely punctate, only with minute setae; setae of anterior and lateral border fine and long but sparse; hypomeron distinctly carinate basally, carina not produced. Scutellum narrow and long, dull, with coarse, dense punctures, narrowly impunctate on basal midline, with minute setae.

Elytra oblong, widest in posterior third, striae weakly impressed, finely and densely punctate, intervals weakly convex, finely and densely punctate, punctures concentrated along striae, penultimate lateral interval with single long and fine, erect setae, otherwise with only very minute setae, otherwise glabrous; epipleural edge wide, ending at widely rounded apical angle of elytra, epipleura densely setose, apical border narrowly membranous, with a fine fringe of microtrichomes (visible at 100×).

Ventral surface moderately shiny, nearly dull, coarsely and densely punctate, metasternum moderately setose; metacoxa glabrous, with a few long setae laterally, posterior margin straight; abdominal sternites finely and densely punctuate, minutely setose, with a transverse row of coarse punctures each bearing a robust, long seta. Mesosternum between mesocoxae half as wide as mesofemur. Ratio of length of metepisternum/metacoxa: 1/1.4. Pygidium strongly convex and shiny, coarsely and densely punctate, with a narrow smooth midline, with a few long erect setae along apical margin.

Legs moderately slender and not very long; femora with two longitudinal rows of setae, finely and sparsely punctate, nearly glabrous; metafemur dull, anterior margin acute, immediately behind anterior edge with a continuously serrated line, punctures and setae of anterior longitudinal row complete, posterior margin in apical half ventrally smooth and not widened, posterior margin smooth dorsally, not serrated, with dense, short setae. Metatibia moderately slender and long, widest at apex, ratio of width/length: 1/3.6; dorsal margin sharply carinate, with three groups of spines, basal group at middle, median one shortly behind middle, and apical group at 4/5 of metatibial length, basally with a few robust but single setae; beside dorsal margin with a continuously serrated line being subparallel with dorsal margin 4/5 of metatibial length; lateral face longitudinally convex, finely and sparsely punctate, sparsely setose, without convex subdorsal longitudinal carina on lateral face; ventral margin finely serrated, with four robust nearly equidistant setae; medial face smooth, apex indistinctly sinuate interiorly near tarsal articulation. Tarsomeres with dense, short setae ventrally, not carinate laterally, smooth dorsally; metatarsomeres with a strongly serrated ventral ridge and a sharp subventral carina immediately beside it, first metatarsomere slightly shorter than following two tarsomeres combined and slightly longer than dorsal tibial spur. Protibia short, tridentate, basal tooth indistinct; anterior claws symmetrical, basal tooth of inner claw sharply truncate at apex.

Aedeagus: Fig. 2I–K. Female unknown.

#### Diagnosis.

The new species differs from all species of the *Neoserica septemlamellata* group with a shiny dorsal surface and in shape of pronotum: its lateral margins are strongly narrowed towards base and concavely sinuate before posterior angles.

#### Etymology.

The new species is named with the composed adjective, *pluri* – (prefix from Latin *plus*, *pluris* - more) and *lamellata* (from Latin *lamellatus* - lamellate)

#### Variation.

Length: 8.3–8.6 mm, length of elytra: 6.9–7.0 mm, width: 5.2–5.8 mm.

### Neoserica (s.l.) bansongchana

Ahrens, Fabrizi & Liu sp. n.

http://zoobank.org/3B1BF1AA-74FF-4BDA-B3F0-00C58BB3982D

http://species-id.net/wiki/Neoserica_bansongchana

Figs 3A–D , 7

#### Type material examined.

Holotype ♂ “Laos, 1.-16.v.1998 Louangphrabang pr. 20°33–4'N, 102°14'E Ban Song Cha (5km W) 1200m, Vít Kubáň leg./ Coll. P. Pacholátko Brno Merhautova 68 Czech Republic/ 176 Sericni: Asia spec.” (CPPB).

#### Description.

Length: 9.7 mm, length of elytra: 6.9 mm, width: 5.7 mm. Body oblong, dark brown, antennal club yellowish brown, dorsal surface dull, except a few long setae on head and sides of elytra nearly glabrous.

Labroclypeus subtrapezoidal, considerably wider than long, widest at base, lateral margins moderately convex and strongly convergent anteriorly, anterior angles strongly rounded, anterior margin weakly sinuate medially, margins moderately reflexed; surface convexly elevated medially and shiny, basis without dull tomentum, punctation dense, small punctures mixed with coarse ones each bearing a long erect seta; frontoclypeal suture distinctly incised, not elevated and distinctly angled medially; smooth area anterior to eye approximately 2.5 times as wide as long; ocular canthus short (length = 1/4 of ocular diameter) and wide, with moderately large punctures and a few long setae. Frons in posterior quarter dull, otherwise moderately shiny, with coarse and fine, dense punctures, densely covered with long erect setae being partly bent posteriorly. Eyes moderately large, ratio diameter/interocular width: 0.63. Antenna with ten antennomeres, club with seven antennomeres, moderately reflexed, 1.5 times as long as remaining antennomeres combined; antennomere 4 subequal to half of length of club, antennomere 3 half as long as pedicellus. Mentum elevated and slightly flattened anteriorly.

Pronotum subrectangular, widest at base, lateral margins evenly convex and moderately convergent anteriorly, anterior angles moderately sharp and distinctly produced, posterior angles blunt, slightly rounded at tip; anterior margin moderately convexly produced medially, with a medially widely incomplete marginal line; surface densely and coarsely punctate, only with minute setae, and a few long erect setae on anterior sides; setae of lateral border fine and moderately dense, those of anterior margin numerous and widely displaced from margin; hypomeron distinctly carinate basally, carina not produced. Scutellum narrow and long, dull, with fine, dense punctures, basally at middle impunctate, with minute setae.

Elytra oblong, widest in posterior third, striae weakly impressed, finely and densely punctate, intervals weakly convex, finely and densely punctate, punctures mainly concentrated along striae, with minute setae, odd intervals in sides and apical declivity with a few erect setae (lacking on disc); epipleural edge wide, ending at widely rounded apical angle of elytra, epipleura densely setose, apical border broadly membranous, with a fine fringe of microtrichomes (visible at 100×).

Ventral surface dull, coarsely and densely punctate, metasternum moderately setose; metacoxa glabrous, with a few long setae laterally, posterior margin straight; abdominal sternites finely and densely punctuate, minutely setose, with a transverse row of coarse punctures each bearing a robust, long seta. Mesosternum between mesocoxae half as wide as mesofemur. Ratio of length of metepisternum/metacoxa: 1/1.56. Pygidium strongly convex and dull, coarsely and densely punctate, with a narrow smooth midline, with dense, long erect setae and minute setae.

Legs moderately slender and not very long; femora with two longitudinal rows of setae, finely and moderately densely punctate, nearly glabrous; metafemur dull, anterior margin acute, immediately behind anterior edge with a continuously serrated line, punctures and setae of anterior longitudinal row complete, posterior margin in apical half ventrally smooth and little widened and smooth, posterior margin smooth dorsally, not serrated, with dense, short setae. Metatibia moderately slender and short, widest at apex, ratio of width/length: 1/2.7; dorsal margin sharply carinate, with three groups of spines, basal group at the middle, median one at two thirds, and apical group at 4/5 of metatibial length, basally with a few robust but single setae; beside dorsal margin with a continuously serrated line being subparallel with dorsal margin 4/5 of metatibial length and ending shortly before metatibial apex; lateral face longitudinally convex, coarsely and moderately densely punctate, with a few robust setae, in apical half punctures less dense, without convex subdorsal longitudinal carina on lateral face; ventral margin finely serrated, with four robust, nearly equidistant setae; medial face smooth, apex indistinctly sinuate interiorly near tarsal articulation. Tarsomeres with dense, short setae ventrally, not carinate laterally, smooth dorsally; metatarsomeres with a strongly serrated ventral ridge and a sharp subventral carina immediately beside it, first metatarsomere as long as following two tarsomeres combined and slightly longer than dorsal tibial spur. Protibia short, distinctly tridentate; anterior claws symmetrical, basal tooth of inner claw sharply truncate at apex.

**Figure 3.**
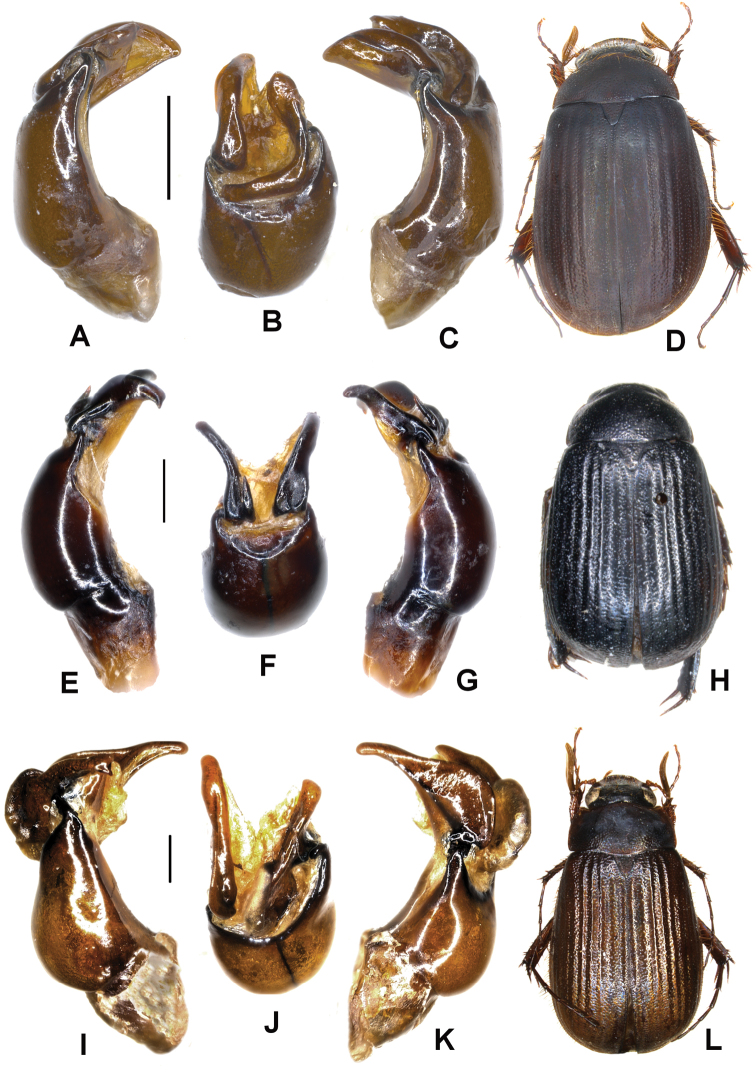
**A–D** *Neoserica bansongchana* Ahrens, Liu & Fabrizi sp. n. (holotype), **E–H** *Neoserica septemfoliata* Moser (lectotype) **I–L** *Neoserica gaoligongshanica* Ahrens, Liu & Fabrizi sp. n. (holotype) **A, E, I** Aedeagus, left side lateral view **C, G, K** Aedeagus, right side lateral view **B, F, J** parameres, dorsal view **D, H, L** Habitus. Scale: 0.5 mm.

Aedeagus: Fig. 3A–C.

#### Diagnosis.

*Neoserica bansongchana* sp. n. differs from *Neoserica takakuwai* sp. n. by the slightly shorter antennal club, and the shorter, more stout parameres.

#### Etymology.

The new species is named according to its type locality Ban Song Cha (Laos).

### Neoserica (s.l.) septemfoliata

Moser, 1915

http://species-id.net/wiki/Neoserica_septemfoliata

Figs 3E–H , 7

Neoserica septemfoliata Moser, 1915b: 381.

#### Type material examined.

Lectotype (here designated): ♂ “Yunnan/ Neoserica septemfoliata Mos. Type Mos.” (ZMHB).

#### Additional material examined.

1 ♂ “Coll. R.I.Sc.N.B. Chine/ Sud Yunnan Tche-Ping-Tcheou/ Coll. P. Guerry Le Moult vendit” (ISNB).

#### Redescription.

Length: 8.4 mm, length of elytra: 6.6 mm, width: 5.2 mm. Body oblong, black, antennal club yellowish brown, dorsal surface shiny, nearly glabrous except a few long setae on head.

Labroclypeus trapezoidal, distinctly wider than long, widest at base, lateral margins weakly convex and convergent anteriorly, anterior angles moderately rounded, anteriorly weakly sinuate medially, margins moderately reflexed; surface slightly convex and shiny, basis without dull tomentum, punctation dense, small punctures mixed with coarse ones each bearing a long erect seta; frontoclypeal suture indistinctly incised, not elevated and distinctly angled medially; smooth area anterior to eye approximately 2.5 times as wide as long; ocular canthus moderately long (length = 1/3 of ocular diameter) and wide, with a few minute punctures and a few long setae. Frons shiny, with coarse and fine, dense punctures, densely covered with long erect setae. Eyes moderately large, ratio diameter/interocular width: 0.64. Antenna with ten antennomeres, club with seven antennomeres, moderately reflexed, 2.5 times as long as remaining antennomeres combined; antennomere 4 subequal to two thirds of length of club, antennomere 3 half as long as pedicellus. Mentum elevated and slightly flattened anteriorly.

Pronotum subrectangular, widest shortly before base, lateral margins evenly convex and moderately convergent anteriorly, slightly narrowed basally, anterior angles sharp and distinctly produced, posterior angles blunt, slightly rounded at tip; anterior margin convexly produced medially, marginal line incomplete medially; surface densely and coarsely punctate, only with minute setae; setae of anterior and lateral border fine and sparse; hypomeron distinctly carinate basally, carina not produced. Scutellum narrow and long, dull, with coarse, dense punctures and minute setae.

Elytra oblong, widest in posterior third, striae weakly impressed, finely and densely punctate, intervals weakly convex, finely and densely punctate, punctures concentrated along striae, with only very minute setae, otherwise glabrous; epipleural edge wide, ending at widely rounded apical angle of elytra, epipleura densely setose, apical border narrowly membranous, with a fine fringe of microtrichomes (visible at 100×).

Ventral surface dull, coarsely and densely punctate, metasternum moderately setose; metacoxa glabrous, with a few long setae laterally, posterior margin straight; abdominal sternites finely and densely punctuate, minutely setose, with a transverse row of coarse punctures each bearing a robust, long seta. Mesosternum between mesocoxae half as wide as mesofemur. Ratio of length of metepisternum/metacoxa: 1/1.49. Pygidium moderately convex and shiny, coarsely and densely punctate, with a narrow smooth midline, with a few long erect setae along apical margin.

Legs moderately slender and not very long; femora with two longitudinal rows of setae, finely and sparsely punctate, nearly glabrous; metafemur dull, anterior margin acute, immediately behind anterior edge with a continuously serrated line, punctures and setae of anterior longitudinal row complete, posterior margin in apical half ventrally smooth and not widened, posterior margin smooth dorsally, not serrated, with dense, short setae. Metatibia moderately slender and long, widest at apex, ratio of width/length: 1/3.2; dorsal margin sharply carinate, with three groups of spines, basal group shortly before middle, median one shortly behind middle, and apical group at 4/5 of metatibial length, basally with a few robust but single setae; beside dorsal margin with a continuously serrated line being subparallel with dorsal margin 4/5 of metatibial length; lateral face longitudinally convex, finely and sparsely punctate, without convex subdorsal longitudinal carina on lateral face; ventral margin finely serrated, with four robust equidistant setae; medial face smooth, apex indistinctly sinuate interiorly near tarsal articulation. Tarsomeres with dense, short setae ventrally, not carinate laterally, smooth dorsally; metatarsomeres with a strongly serrated ventral ridge and a sharp subventral carina immediately beside it, first metatarsomere distinctly shorter than following two tarsomeres combined and slightly longer than dorsal tibial spur. Protibia short, tridentate, basal tooth blunt; anterior claws symmetrical, basal tooth of inner claw sharply truncate at apex.

Aedeagus: Fig. 3E–G. Female unknown.

#### Remarks.

We designate here the lectotype for the species, since in the original description it is not mentioned on how many specimens it was based.

### Neoserica (s.l.) gaoligongshanica

Ahrens, Fabrizi & Liu sp. n.

http://zoobank.org/D81287E8-AEDF-4678-AF2E-84D259899683

http://species-id.net/wiki/Neoserica_gaoligongshanica

Figs 3I–L , 7

#### Type material examined.

Holotype ♂ “China, Yunnan prov.; Gaoligongshan mts.; 90km W of Baoshan; S. Bečvář leg.; 26-29.v.1995/ Coll. P. Pacholátko Brno Merhautova 68 Czech Republic/ 714 Sericni: Asia spec.” (CPPB). Paratypes: 8 ♂♂, 3 ♀♀ “China, W Yunnan prov., mts. 60Km E Tengchong, 2300m 14.-19.v.2006, S. Murzin & I. Shokhin leg./ Coll. P. Pacholátko Brno Merhautova 68 Czech Republic” (CPPB, ZFMK).

#### Description.

Length: 10.5 mm, length of elytra: 7.3 mm, width: 5.9 mm. Body oblong, dark reddish brown, antennal club yellowish brown, dorsal surface except dull pronotum moderately shiny, except a few long setae on head and sides of elytra nearly glabrous.

Labroclypeus trapezoidal, distinctly wider than long, widest at base, lateral margins weakly convex and convergent anteriorly, anterior angles moderately rounded, anteriorly very weakly sinuate medially, margins moderately reflexed; surface slightly convex and shiny, basis without dull tomentum, punctation dense, small punctures mixed with coarse ones each bearing a long erect seta; frontoclypeal suture indistinctly incised, slightly elevated and distinctly angled medially; smooth area anterior to eye approximately twice as wide as long; ocular canthus moderately long (length = 1/3 of ocular diameter) and wide, with a few minute punctures and a few long setae. Frons shiny, in posterior third dull, with coarse and fine, dense punctures, densely covered with long erect setae. Eyes moderately large, ratio diameter/interocular width: 0.65. Antenna with ten antennomeres, club with seven antennomeres, moderately reflexed, 2.5 times as long as remaining antennomeres combined; antennomere 4 subequal to two thirds of length of club, antennomere 3 half as long as pedicellus. Mentum elevated and slightly flattened anteriorly.

Pronotum moderately transverse, subrectangular, widest a quarter before base, weakly shiny, on disc with dull tomentum, lateral margins evenly convex and moderately convergent anteriorly, slightly narrowed basally, anterior angles moderately sharp and distinctly produced, posterior angles blunt, slightly rounded at tip; anterior margin convexly produced medially, marginal line incomplete medially; surface densely and coarsely punctate, only with minute setae; setae of anterior and lateral border fine and sparse; hypomeron distinctly carinate basally, carina not produced. Scutellum narrow and long, dull, with coarse, dense punctures and minute setae.

Elytra oblong, widest in posterior third, striae weakly impressed, finely and densely punctate, intervals weakly convex, finely and densely punctate, punctures except on second interval concentrated along striae, with only very minute setae, otherwise glabrous, penultimate lateral interval with single, long erect setae, ultimate lateral interval with short, fine, adjacent setae instead of minute ones; epipleural edge wide, ending at widely rounded apical angle of elytra, epipleura densely setose, apical border narrowly membranous, with a fine fringe of microtrichomes (visible at 100×).

Ventral surface dull, coarsely and densely punctate, metasternum moderately setose; metacoxa glabrous, with a few long setae laterally, posterior margin slightly concave; abdominal sternites finely and densely punctuate, minutely setose, with a transverse row of coarse punctures each bearing a more robust, long seta. Mesosternum between mesocoxae half as wide as mesofemur. Ratio of length of metepisternum/ metacoxa: 1/1.34. Pygidium strongly convex and shiny, coarsely and densely punctate, with a narrow smooth midline, with a few long erect setae on apical half.

Legs moderately slender and not very long; femora with two longitudinal rows of setae, finely and sparsely punctate, nearly glabrous; metafemur dull, anterior margin acute, immediately behind anterior edge with a continuously serrated line, punctures and setae of anterior longitudinal row complete, posterior margin in apical half ventrally smooth and not widened, posterior margin smooth dorsally, not serrated, with dense, short setae. Metatibia moderately slender and long, widest at apex, ratio of width/length: 1/3.5; dorsal margin sharply carinate, with three groups of spines, basal group shortly before middle, median one shortly behind middle, and apical group at 4/5 of metatibial length, basally with a few robust but single setae; beside dorsal margin with a continuously serrated line being subparallel with dorsal margin 4/5 of metatibial length; lateral face longitudinally convex, finely and sparsely punctate, without convex subdorsal longitudinal carina on lateral face; ventral margin finely serrated, with four robust setae with the posterior one being more distant from the others; medial face smooth, apex concavely sinuate interiorly near tarsal articulation. Tarsomeres with dense, short setae ventrally, not carinate laterally, smooth dorsally; metatarsomeres with a strongly serrated ventral ridge and a sharp subventral carina immediately beside it, first metatarsomere slightly shorter than following two tarsomeres combined and slightly longer than dorsal tibial spur. Protibia short, tridentate, but basal tooth rather indistinct; anterior claws symmetrical, basal tooth of inner claw sharply truncate at apex.

Aedeagus: Fig. 3I–K.

#### Diagnosis.

*Neoserica gaoligongshanica* sp. n. is in external shape most similar to *Neoserica septemfoliata*, but it differs by the dull pronotum, the larger body size, and the shape of male copulatory organ: the phallobasis is wider, strongly asymmetric apically, with the parameres being strongly dorsoventrally produced having large basal lobes fused with the rest of the paramere.

#### Etymology.

The new species is named according its type locality, Gaoligongshan.

#### Variation.

Length: 10.2–11.3 mm, length of elytra: 7.3–8.0 mm, width: 5.9–6.0 mm. Female: Antennal club composed of five antennomeres, slightly longer than remaining antennomeres combined, first joint of club a quarter to one third of length of club, 5th antennomere slightly transversely produced; eyes slightly smaller than in male (ratio diameter/interocular width: 0.58).

### Neoserica (s.l.) daweishanica

Ahrens, Fabrizi & Liu sp. n.

http://zoobank.org/AF9F30F7-1886-4AF5-8D07-497A9A3542FF

http://species-id.net/wiki/Neoserica_daweishanica

Figs 4A–D , 6

#### Type material examined.

Holotype ♂ “Mt. Daweishan, Pingbian, Yunnan, 19.VI.1956, 1500m, leg. Huang Keren etc.” (IZAS). Paratype. 1 ♂ “Mt. Daweishan, Pingbian, Yunnan, 23.VI.1956, 1300m, leg. Huang Keren etc.” (ZFMK).

#### Description.

Length: 9.5 mm, length of elytra: 6.9 mm, width: 5.2 mm. Body oblong, reddish brown, antennal club yellowish brown, dorsal surface moderately shiny, nearly glabrous except a few long setae on head.

Labroclypeus trapezoidal, distinctly wider than long, widest at base, lateral margins straight and distinctly convergent anteriorly, anterior angles moderately rounded, anteriorly weakly sinuate medially, margins moderately reflexed; surface slightly convex and shiny, basis without dull tomentum, punctation dense, small punctures mixed with coarse ones each bearing a long erect seta; frontoclypeal suture distinctly incised, slightly elevated and distinctly angled medially; smooth area anterior to eye approximately 2.5 times as wide as long; ocular canthus moderately long (length = 1/3 of ocular diameter) and wide, with a few minute punctures and a few long setae. Frons shiny, with coarse and fine, dense punctures, densely covered with long erect setae. Eyes moderately large, ratio diameter/interocular width: 0.71. Antenna with ten antennomeres, club with seven antennomeres, moderately reflexed, twice as long as remaining antennomeres combined; antennomere 4 subequal to half length of club, antennomere 3 half as long as pedicellus. Mentum elevated and slightly flattened anteriorly.

Pronotum moderately transverse, subrectangular, widest at base, lateral margins evenly weakly convex and moderately convergent anteriorly, anterior angles sharp and distinctly produced, posterior angles nearly blunt but strongly rounded at tip; anterior margin convexly produced medially, marginal line incomplete medially; surface densely and coarsely punctate, only with minute setae; setae of anterior and lateral border fine and sparse; hypomeron distinctly carinate basally, carina not produced. Scutellum narrow and long, dull, with coarse, dense punctures and minute setae.

Elytra oblong, widest in posterior third, striae weakly impressed, finely and densely punctate, intervals moderately convex, finely and densely punctate, punctures concentrated along striae, with only very minute setae, penultimate lateral interval with a few single fine and long erect setae, otherwise glabrous; epipleural edge wide, ending at widely rounded apical angle of elytra, epipleura densely setose, apical border narrowly membranous, with a fine fringe of microtrichomes (visible at 100×).

Ventral surface dull, coarsely and densely punctate, metasternum moderately setose; metacoxa glabrous, with a few long setae laterally, posterior margin straight; abdominal sternites finely and densely punctuate, minutely setose, with a transverse row of coarse punctures each bearing a robust, long seta. Mesosternum between mesocoxae half as wide as mesofemur. Ratio of length of metepisternum/metacoxa: 1/1.4. Pygidium moderately convex and shiny, coarsely and densely punctate, with a narrow smooth midline, with a few long erect setae along apical margin.

Legs moderately slender and not very long; femora with two longitudinal rows of setae, finely and sparsely punctate, nearly glabrous; metafemur dull, anterior margin acute, immediately behind anterior edge with a continuously serrated line, punctures and setae of anterior longitudinal row complete, posterior margin in apical half ventrally smooth and not widened, posterior margin smooth dorsally, not serrated, with dense, short setae. Metatibia moderately slender and long, widest at apex, ratio of width/length: 1/3.3; dorsal margin sharply carinate, with three groups of spines, basal group shortly before middle, median one shortly behind middle, and apical group at 4/5 of metatibial length, basally with a few robust but single setae; beside dorsal margin with a continuously serrated line being subparallel with dorsal margin 4/5 of metatibial length; lateral face longitudinally convex, finely and sparsely punctate, with a few single setae, without convex subdorsal longitudinal carina on lateral face; ventral margin finely serrated, with four robust equidistant setae; medial face smooth, apex indistinctly sinuate interiorly near tarsal articulation. Tarsomeres with dense, short setae ventrally, not carinate laterally, smooth dorsally; metatarsomeres with a strongly serrated ventral ridge and a sharp subventral carina immediately beside it, first metatarsomere slightly longer than dorsal tibial spur, subsequent tarsomeres lacking in holotype. Protibia short, tridentate; anterior claws symmetrical, basal tooth of inner claw sharply truncate at apex.

**Figure 4.**
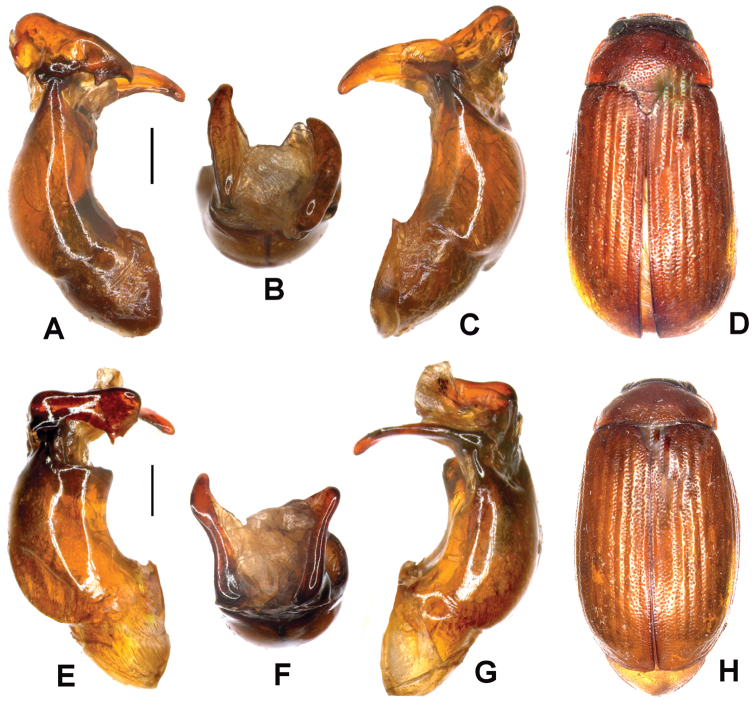
**A–D** *Neoserica daweishanica* Ahrens, Liu & Fabrizi sp. n. (holotype) **E–H** *Neoserica jiulongensis* Ahrens, Liu & Fabrizi sp. n. (holotype) **A, E** Aedeagus, left side lateral view **C, G** Aedeagus, right side lateral view **B, F** parameres, dorsal view **D, H** Habitus. Scale: 0.5 mm.

Aedeagus: Fig. 4A–C. Female unknown.

#### Diagnosis.

The new species is in external appearance rather similar to *Neoserica septemfoliata*; it differs in the shape of left paramere (dorsal view), which is strongly widened basally (lateral view) having a sharp ventral tooth at apex. Furthermore its antennal club is in male only twice as long as the remaining antennomeres combined (instead of 2.5 times as in *Neoserica septemfoliata*).

#### Etymology.

The new species is named after its type locality, Mt. Daweishan.

#### Variation.

Length: 8.6–9.5 mm, length of elytra: 6.5–6.9 mm, width: 5.2 mm. First metatarsomere slightly shorter than following two tarsomeres combined.

### Neoserica (s.l.) jiulongensis

Ahrens, Fabrizi & Liu sp. n.

http://zoobank.org/CBAB425A-AD3B-44D8-9842-5866C0A5FB5B

http://species-id.net/wiki/Neoserica_jiulongensis

Figs 4E–H , 6

#### Type material examined.

Holotype ♂ “Jiulong, Sichuan, 26.VI.1979, leg. Collecting Group” (IZAS).

#### Description.

Length: 9.4 mm, length of elytra: 6.9 mm, width: 5.0 mm. Body oblong, reddish brown, antennal club yellowish brown, dorsal surface moderately shiny, nearly glabrous except a few long setae on head.

Labroclypeus trapezoidal, distinctly wider than long, widest at base, lateral margins weakly convex and convergent anteriorly, anterior angles moderately rounded, anteriorly weakly sinuate medially, margins moderately reflexed; surface slightly convex and shiny, basis without dull tomentum, punctation dense, small punctures mixed with coarse ones each bearing a long erect seta; frontoclypeal suture indistinctly incised, not elevated and distinctly angled medially; smooth area anterior to eye approximately 2.5 times as wide as long; ocular canthus moderately long (length = 1/3 of ocular diameter) and wide, with a few minute punctures and one or two long terminal setae. Frons shiny, with fine, dense punctures, beside eyes and behind frontoclypeal suture with long erect setae, on disc only a few slightly shorter setae. Eyes moderately large, ratio diameter/interocular width: 0.61. Antenna with ten antennomeres, club with seven antennomeres, moderately reflexed, twice as long as remaining antennomeres combined; antennomere 4 subequal to half length of club, antennomere 3 half as long as pedicellus. Mentum elevated and slightly flattened anteriorly.

Pronotum subrectangular, widest shortly before base, lateral margins evenly convex and moderately convergent anteriorly, slightly narrowed basally, anterior angles sharp and distinctly produced, posterior angles blunt, slightly rounded at tip; anterior margin moderately convexly produced medially, marginal line incomplete medially; surface densely and coarsely punctate, only with minute setae; setae of anterior and lateral border fine and sparse; hypomeron distinctly carinate basally, carina not produced. Scutellum narrow and long, dull, with coarse, dense punctures and minute setae.

Elytra oblong, widest shortly behind middle, striae weakly impressed, finely and densely punctate, intervals weakly convex, finely and densely punctate, punctures concentrated along striae, with only very minute setae, penultimate lateral intervals with single, long setae, otherwise glabrous; epipleural edge wide, ending at widely rounded apical angle of elytra, epipleura densely setose, apical border narrowly membranous, with a fine fringe of microtrichomes (visible at 100×).

Ventral surface dull, coarsely and densely punctate, metasternum moderately setose; metacoxa glabrous, with a few long setae laterally, posterior margin straight; abdominal sternites finely and densely punctuate, minutely setose, with a transverse row of coarse punctures each bearing a robust, long seta. Mesosternum between mesocoxae half as wide as mesofemur. Ratio of length of metepisternum/metacoxa: 1/1.35. Pygidium strongly convex apically and shiny, finely and very densely punctate, with a narrow smooth midline, with a few long erect setae along apical margin.

Legs moderately slender and not very long; femora with two longitudinal rows of setae, finely and sparsely punctate, nearly glabrous; metafemur dull, anterior margin acute, immediately behind anterior edge with a continuously serrated line, punctures and setae of anterior longitudinal row complete, posterior margin in apical half ventrally smooth and not widened, posterior margin smooth dorsally, not serrated, with dense, short setae. Metatibia moderately slender and long, widest at apex, ratio of width/length: 1/3.4; dorsal margin sharply carinate, with three groups of spines, basal group shortly before middle, median one shortly behind middle, and apical group at 4/5 of metatibial length, basally with a few robust but single setae; beside dorsal margin with a continuously serrated line being subparallel with dorsal margin 4/5 of metatibial length; lateral face longitudinally convex, finely and sparsely punctate, with sparse fine setae, without convex subdorsal longitudinal carina on lateral face; ventral margin finely serrated, with four robust equidistant setae; medial face smooth, apex indistinctly sinuate interiorly near tarsal articulation. Tarsomeres with dense, short setae ventrally, not carinate laterally, smooth dorsally; metatarsomeres with a strongly serrated ventral ridge and a sharp subventral carina immediately beside it, first metatarsomere distinctly shorter than following two tarsomeres combined and slightly longer than dorsal tibial spur. Protibia short, tridentate, basal tooth blunt; anterior claws symmetrical, basal tooth of inner claw sharply truncate at apex.

Aedeagus: Fig. 4E–G. Female unknown.

#### Diagnosis.

The new species shares with *Neoserica plurilamellata* sp. n. a ventroapically produced phallobasis. It can be easily distinguished by the evenly narrowed posterior lateral margins of pronotum (being not deeply concavely sinuate as in *Neoserica plurilamellata* sp. n.) and the left paramere, which is apically abruptly widened.

#### Etymology.

The new species is named after its type locality, Jiulong.

### Neoserica (s.l.) changrae

Ahrens, 2004

Fig. 6

Neoserica changrae Ahrens, 2004: 167.

#### Distribution.

Bhutan: so far only known from type locality, Changra, 18 km S Tongsa 1900 m [27°24'N, 90°27'E].

### Neoserica (s.l.) crenatolineata

Ahrens & Fabrizi, 2009

Fig. 6

Neoserica crenatolineata Ahrens & Fabrizi, 2009: 262.

#### Distribution.

Arunachal Pradesh (India): so far only known from type locality, Dirang vicinity [27°21–23'N, 92°13–16'E].

### Neoserica (s.l.) sapaensis

Ahrens, Fabrizi & Liu sp. n.

http://zoobank.org/91F3EE70-8468-4AFF-9ACB-B6B03D1D492F

http://species-id.net/wiki/Neoserica_sapaensis

Figs 5A–D , 7

#### Type material examined.

Holotype ♂ “N-Vietnam Sa Pa env., Lao Cai Prov. 22°19'52"N, 103°50'35"E 1630–1680m 23.–27.V.1999 leg. Fabrizi, Jäger, Ahrens” (ZFMK). Paratypes: 13 ♂♂, 6 ♀♀ “N-Vietnam Sa Pa env., Lao Cai Prov. 22°19'52"N, 103°50'35"E, 1630–1680m 23.–27.V.1999 leg. Fabrizi, Jäger, Ahrens” (ZFMK), 3 ♂♂ “N.-Vietnam Fan Si Pan near Sapa, 1500–1950m 17.–30.VI.1999 A. Kallies leg.” (ZFMK), 1 ♂, 2 ♀♀ “N. Vietnam: Lao Cai Prov. Sa Pa 2-4/VII/1997 collr. C.L. Li” (ZFMK), 2 ♂♂ “Vietnam N (Sa Pa) Lao Cai Prov., 250km from Hanoi bearing 31°, Sa Pa vill. env. Hoang Lien Son Nat. Res. 16.–20.VI.1998 1250m leg. A. Napolov” (CNAR), 1 ♂ “Vietnam N (Sa Pa) Lao Cai Prov., 250km from Hanoi bearing 31°, Sa Pa vill. env. Hoang Lien Son Nat. Res. 27.V.–15.VI.1995 1250m leg. A. Napolov” (CNAR), 1 ♂ N Vietnam (Tonkin) pr. Vinh Phu 1990 Tam Dao 17.–21.V. P. Pacholátko leg./ VS92” (CPPB), 1 ♂ “N Vietnam, 21,35N 106,30E 52km SW of Lang Son, 27.iv.–6.v.1996, 370m Pacholátko & Dembický leg./ VS106” (CPPB), 1 ♂ “Chine-Yunnan Res. Huanglian Shan 22°54'N, 102°18'E alt. 1900M/ Museum Paris 19.VI.2001 Deuve, Mantilleri, Rougerie & Tian leg.” (MNHN), 1 ♂ “Yunnan, Yakou, 2012-V-11” (IZAS), 2 ♂♂ “Hetouzhai, Jinping, Yunnan, 12.V.1956, 1600-1700m, leg. Huang Keren etc.” (IZAS), 1 ♂ “Hetouzhai, Jinping, Yunnan, 14.V.1956, 1700m, light trap, leg. Huang Keren etc.” (IZAS), 1 ♂ “Hetouzhai, Jinping, Yunnan, 10.V.1956, 2000m, leg. Huang Keren” (IZAS), 1 ♂ “Hetouzhai, Jinping, Yunnan, 9.V.1956, 1700m, leg. Huang Keren etc.” (IZAS).

#### Description.

Length: 9.9 mm, length of elytra: 6.8 mm, width: 5.3 mm. Body oblong, dark reddish brown, antennal club yellowish brown, dorsal surface dull, nearly glabrous except a few long setae on head.

Labroclypeus trapezoidal, distinctly wider than long, widest at base, lateral margins nearly straight and strongly convergent anteriorly, anterior angles strongly rounded, anterior margin very weakly sinuate medially, margins moderately reflexed; surface flat and shiny, basis without dull tomentum, punctation dense, small punctures mixed with coarse ones each bearing a long erect seta; frontoclypeal suture distinctly incised, not elevated and distinctly angled medially; smooth area anterior to eye approximately 2.5 times as wide as long; ocular canthus moderately long (length = 1/3 of ocular diameter) and wide, with minute punctures and a few long setae. Frons dull, anterior quarter shiny, with coarse and fine, dense punctures, densely covered with long erect setae. Eyes large, ratio diameter/interocular width: 0.74. Antenna with ten antennomeres, club with seven antennomeres, moderately reflexed, 2.5 times as long as remaining antennomeres combined; antennomere 4 subequal to two thirds of length of club, antennomere 3 half as long as pedicellus. Mentum elevated and slightly flattened anteriorly.

Pronotum subrectangular, widest at base, lateral margins nearly straight and subparallel in basal half, evenly convex and moderately convergent in anterior half, anterior angles moderately sharp and distinctly produced, posterior angles blunt, slightly rounded at tip; anterior margin convexly produced medially, marginal line incomplete medially; surface densely and coarsely punctate, only with minute setae; setae of anterior and lateral border fine and moderately dense; hypomeron distinctly carinate basally, carina not produced. Scutellum narrow and long, dull, with fine, dense punctures and minute setae.

Elytra oblong, widest in posterior third, striae weakly impressed, finely and densely punctate, intervals weakly convex, finely and densely punctate, punctures concentrated along striae, with minute setae, odd intervals with a few erect setae (partially lacking on disc); epipleural edge wide, ending at widely rounded apical angle of elytra, epipleura densely setose, apical border narrowly membranous, with a fine fringe of microtrichomes (visible at 100×).

Ventral surface dull, coarsely and densely punctate, metasternum moderately setose; metacoxa glabrous, with a few long setae laterally, posterior margin straight; abdominal sternites finely and densely punctuate, minutely setose, with a transverse row of coarse punctures each bearing a robust, long seta. Mesosternum between mesocoxae half as wide as mesofemur. Ratio of length of metepisternum/metacoxa: 1/1.31. Pygidium strongly convex and dull, coarsely and densely punctate, with a narrow smooth midline, with dense, long erect setae and minute setae.

Legs moderately slender and not very long; femora with two longitudinal rows of setae, finely and moderately densely punctate, nearly glabrous; metafemur dull, anterior margin acute, immediately behind anterior edge with a continuously serrated line, punctures and setae of anterior longitudinal row complete, posterior margin in apical half ventrally smooth and not widened, posterior margin smooth dorsally, not serrated, with dense, short setae. Metatibia moderately slender and long, widest at apex, ratio of width/length: 1/2.9; dorsal margin sharply carinate, with three groups of spines, basal group shortly before middle, median one shortly behind middle, and apical group at 4/5 of metatibial length, basally with a few robust but single setae; beside dorsal margin with a continuously serrated line being subparallel with dorsal margin 4/5 of metatibial length; lateral face longitudinally convex, finely and moderately densely punctate, without convex subdorsal longitudinal carina on lateral face; ventral margin finely serrated, with four robust equidistant setae; medial face smooth, apex indistinctly sinuate interiorly near tarsal articulation. Tarsomeres with dense, short setae ventrally, not carinate laterally, smooth dorsally; metatarsomeres with a strongly serrated ventral ridge and a sharp subventral carina immediately beside it, first metatarsomere distinctly shorter than following two tarsomeres combined and slightly longer than dorsal tibial spur. Protibia short, tridentate, basal tooth blunt; anterior claws symmetrical, basal tooth of inner claw sharply truncate at apex.

**Figure 5.**
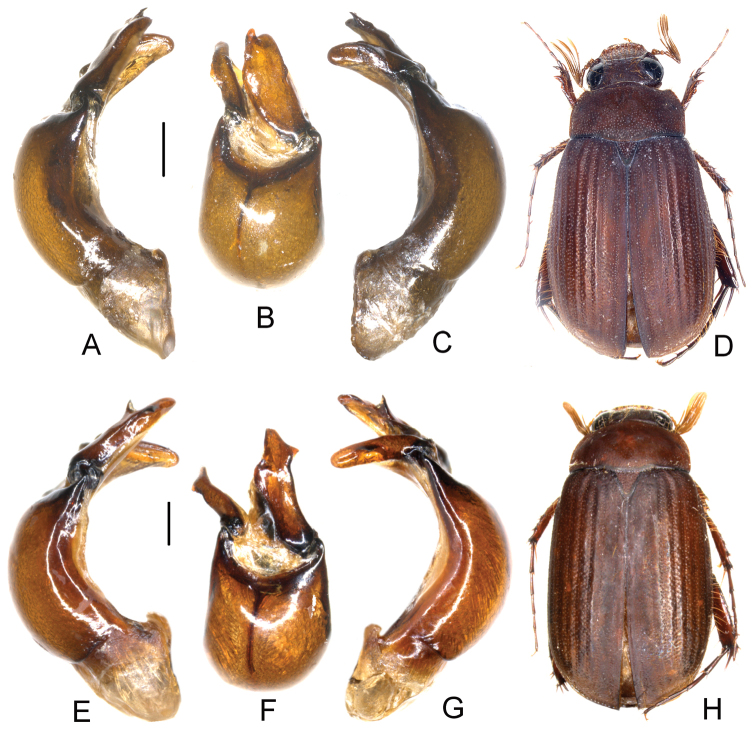
**A–D** *Neoserica sapaensis* Ahrens, Liu & Fabrizi sp. n. (holotype) **E–H** *Neoserica igori* Ahrens, Liu & Fabrizi sp. n. (holotype) **A, E** Aedeagus, left side lateral view **C, G** Aedeagus, right side lateral view **B, F** parameres, dorsal view **D, H** Habitus. Scale: 0.5 mm.

Aedeagus: Fig. 5A–C.

#### Diagnosis.

The new species is quite similar to *Neoserica septemlamellata* in external morphology. It may be distinguished by the lacking dense dorsal pilosity on pronotum and elytra, and by the shape of male copulation organ: left paramere much wider, without basal lobe, right paramere not widened apically but with a lateral tooth at the middle.

#### Etymology.

*Neoserica sapaensis* sp. n. is named according to its type locality Sa Pa (Vietnam).

#### Variation.

Length: 8.9–9.9 mm, length of elytra: 6.4–6.8 mm, width: 4.9–5.3 mm. Female: Antennal club composed of five antennomeres, slightly longer than remaining antennomeres combined, first joint of club a quarter to one third of length of club, 5th antennomere slightly transversely produced; eyes slightly smaller than in male (ratio diameter/interocular width: 0.62).

### Neoserica (s.l.) igori

Ahrens, Fabrizi & Liu sp. n.

http://zoobank.org/6282FD81-D221-4952-A48F-2959D23B466A

http://species-id.net/wiki/Neoserica_igori

Figs 5E–H , 6

#### Type material examined.

Holotype: ♂ “China, N.Yunnan, env. Xiaguan, 2400m, 29.vii.2002, leg. S. Murzin, I. Shokhin/ Coll. P. Pacholátko Brno Merhautova 68 Czech Republic/ 821 Sericini: Asia spec.” (CPPB). Paratypes: 1 ♂, 1 ♀ “China, N.Yunnan, env. Xiaguan, 2400m, 29.vii.2002, leg. S. Murzin, I. Shokhin/ Coll. P. Pacholátko Brno Merhautova 68 Czech Republic ” (CPPB, ZFMK), 4 ♂♂ “China, W. Yunnan, env. Baoshan, 2500m, 2.–3.viii.2002, leg. S. Murzin, I. Shokhin/ Coll. P. Pacholátko Brno Merhautova 68 Czech Republic ” (CPPB, ZFMK), 1 ♂ “Mt. Heishan, Longxin, Longling, Yunnan, 23-25.VII.2008, leg. Xu Jishan, Gao Zhenhua” (HBUM).

#### Description.

Length: 9.0 mm, length of elytra: 6.5 mm, width: 4.9 mm. Body oblong, dark reddish brown, antennal club yellowish brown, dorsal surface dull, nearly glabrous except a few long setae on head.

Labroclypeus trapezoidal, distinctly wider than long, widest at base, lateral margins nearly straight and strongly convergent anteriorly, anterior angles strongly rounded, anterior margin nearly straight, margins moderately reflexed; surface flat and shiny, basis without dull tomentum, punctation dense, small punctures mixed with coarse ones each bearing a long erect seta; frontoclypeal suture distinctly incised, not elevated and distinctly angled medially; smooth area anterior to eye approximately 2.5 times as wide as long; ocular canthus moderately long (length = 1/3 of ocular diameter) and wide, with minute punctures and a few long setae. Frons dull, anterior quarter shiny, with coarse and fine, dense punctures, densely covered with long erect setae. Eyes large, ratio diameter/interocular width: 0.74. Antenna with ten antennomeres, club with seven antennomeres, moderately reflexed, 2.5times as long as remaining antennomeres combined; antennomere 4 subequal to two thirds of length of club, antennomere 3 half as long as pedicellus. Mentum elevated and slightly flattened anteriorly.

Pronotum subrectangular, widest at base, lateral margins nearly straight and subparallel in basal half, evenly convex and moderately convergent in anterior half, anterior angles moderately sharp and distinctly produced, posterior angles blunt, slightly rounded at tip; anterior margin convexly produced medially, marginal line incomplete medially; surface densely and coarsely punctate, only with minute setae; setae of anterior and lateral border fine and moderately dense; hypomeron distinctly carinate basally, carina not produced. Scutellum narrow and long, dull, with fine, dense punctures and minute setae.

Elytra oblong, widest in posterior third, striae weakly impressed, finely and densely punctate, intervals weakly convex, finely and densely punctate, punctures concentrated along striae, with minute setae, odd intervals with a few erect setae (partially lacking on disc); epipleural edge wide, ending at widely rounded apical angle of elytra, epipleura densely setose, apical border narrowly membranous, with a fine fringe of microtrichomes (visible at 100×).

Ventral surface dull, coarsely and densely punctate, metasternum moderately setose; metacoxa glabrous, with a few long setae laterally, posterior margin straight; abdominal sternites finely and densely punctuate, minutely setose, with a transverse row of coarse punctures each bearing a robust, long seta. Mesosternum between mesocoxae half as wide as mesofemur. Ratio of length of metepisternum/metacoxa: 1/1.33. Pygidium strongly convex and dull, coarsely and densely punctate, with a narrow smooth midline, with dense, long erect setae and minute setae.

Legs moderately slender and not very long; femora with two longitudinal rows of setae, finely and moderately densely punctate, nearly glabrous; metafemur dull, anterior margin acute, immediately behind anterior edge with a continuously serrated line, punctures and setae of anterior longitudinal row complete, posterior margin in apical half ventrally smooth and not widened, posterior margin smooth dorsally, not serrated, with dense, short setae. Metatibia moderately slender and long, widest at apex, ratio of width/length: 1/3.1; dorsal margin sharply carinate, with three groups of spines, basal group shortly before middle, median one shortly behind middle, and apical group at 4/5 of metatibial length, basally with a few robust but single setae; beside dorsal margin with a continuously serrated line being subparallel with dorsal margin 4/5 of metatibial length; lateral face longitudinally convex, finely and moderately densely punctate, without convex subdorsal longitudinal carina on lateral face; ventral margin finely serrated, with four robust equidistant setae; medial face smooth, apex indistinctly sinuate interiorly near tarsal articulation. Tarsomeres with dense, short setae ventrally, not carinate laterally, smooth dorsally; metatarsomeres with a strongly serrated ventral ridge and a sharp subventral carina immediately beside it, first metatarsomere distinctly shorter than following two tarsomeres combined and slightly longer than dorsal tibial spur. Protibia short, tridentate, basal tooth blunt; anterior claws symmetrical, basal tooth of inner claw sharply truncate at apex.

Aedeagus: Fig. 5E–G.

#### Diagnosis.

The new species is similar to *Neoserica sapaensis* sp. n. in external and genital morphology. It may be distinguished by the presence of a lateral external tooth at apical external margin of the left paramere.

#### Etymology.

The new species is named after one of its collectors, Igor Shokhin.

#### Variation.

Length: 8.6–9.5 mm, length of elytra: 6.5–7.2 mm, width: 4.9–5.2 mm. Female: Antennal club with five antennomeres, short, as long as remaining antennomeres combined, first joint of club half as long as club; eyes smaller than in male (ratio diameter/interocular width: 0.58).

**Figure 6.**
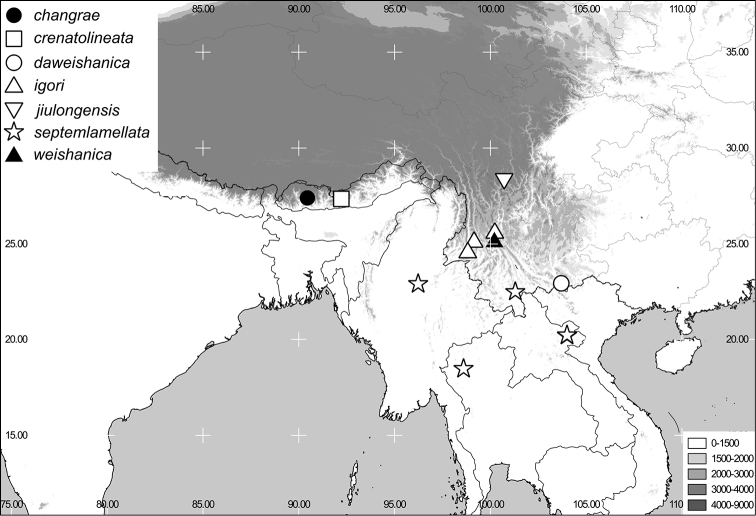
Distribution of the species of the *Neoserica* (s.l.) *septemlamellata* group: *Neoserica changrae*, *Neoserica crenatolineata*, *Neoserica daweishanica*, *Neoserica igori*, *Neoserica jiulongensis*, *Neoserica septemlamellata*, *Neoserica weishanica*.

**Figure 7.**
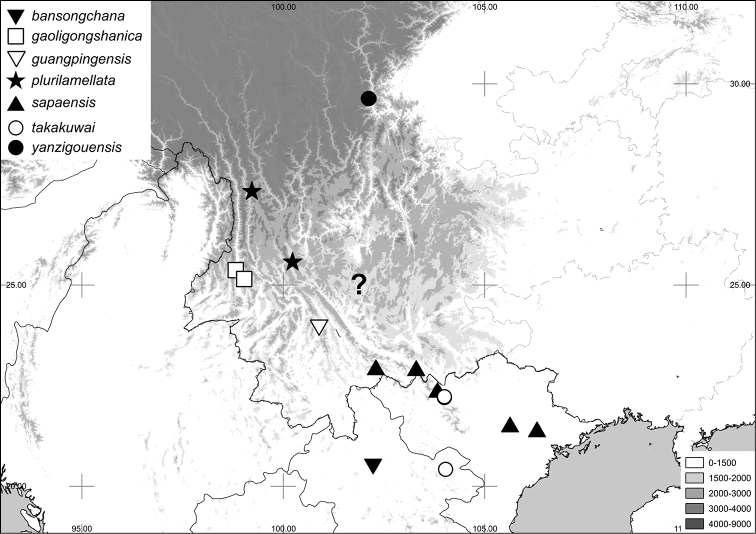
Distribution of the species of the *Neoserica* (s.l.) *septemlamellata* group: *Neoserica bansongchana*, *Neoserica gaoligongshanica*, *Neoserica guangpingensis*, *Neoserica plurilamellata*, *Neoserica sapaensis*, *Neoserica takakuwai*, *Neoserica yanzigouensis*."?" stands for the not localised records of *Neoserica septemfoliata* from Yunnan.

## Supplementary Material

XML Treatment for Neoserica (s.l.) septemlamellata

XML Treatment for Neoserica (s.l.) weishanica

XML Treatment for Neoserica (s.l.) takakuwai

XML Treatment for Neoserica (s.l.) yanzigouensis

XML Treatment for Neoserica (s.l.) guangpingensis

XML Treatment for Neoserica (s.l.) plurilamellata

XML Treatment for Neoserica (s.l.) bansongchana

XML Treatment for Neoserica (s.l.) septemfoliata

XML Treatment for Neoserica (s.l.) gaoligongshanica

XML Treatment for Neoserica (s.l.) daweishanica

XML Treatment for Neoserica (s.l.) jiulongensis

XML Treatment for Neoserica (s.l.) changrae

XML Treatment for Neoserica (s.l.) crenatolineata

XML Treatment for Neoserica (s.l.) sapaensis

XML Treatment for Neoserica (s.l.) igori
